# Theoretical investigation of electrocatalytic activity of Pt-free dual atom-doped graphene for O_2_ reduction in an alkaline solution

**DOI:** 10.1038/s41598-024-61223-y

**Published:** 2024-06-20

**Authors:** Tahereh Jangjooye Shaldehi, Soosan Rowshanzamir

**Affiliations:** Hydrogen & Fuel Cell Research Laboratory, School of Chemical, Petroleum and Gas Engineering, Iran University of Science and Technology, Narmak, 16846-13114 Tehran Iran

**Keywords:** Density functional theory, Oxygen reduction reaction, Dual atom electrocatalyst, Alkaline solution, Analytical chemistry, Catalysis, Chemical engineering, Electrochemistry, Energy, Materials chemistry, Theoretical chemistry

## Abstract

Non-precious electrocatalysts as the alternative to Pt have become a hot research area in the last decade due to the suitable catalytic activity in Oxygen reduction reaction (ORR) in electrochemical systems. In this work, the density functional theory calculations were investigated to explore the activity of Fe, Cu, and Fe-Cu atoms supported by N-doped graphene as the ORR electrocatalyst for Oxygen-depolarized cathodes (ODCs). To this end, the ORR mechanism was surveyed in detail in the gas and solvent phases. The results show that the solvent phase leads to a higher overpotential and thermodynamic limiting potential. According to the density of states curves, there are strong interactions between metal atom and substrate that can effectively tune the electronics of catalysts. Bader's analysis confirms that, in addition to the single metal atoms, nitrogen atoms have also played a critical role in charge transfer between substrates and oxygen molecules in ORR. It is also predicted that Fe-Cu@NC SAC exhibits the highest catalytic activity which is consistent with thermodynamic limiting potential and theoretical overpotential of  − 0.26 and  0.66 (V vs. SHE), respectively, indicating that this type of catalyst may be a suitable candidate instead of precious metals in oxygen-depolarized cathodes in electrochemical devices.

## Introduction

Oxygen-depolarized cathodes (ODCs) are indispensable components within a variety of electrochemical systems, encompassing metal-air batteries, fuel cells, and electrolyzers^1–4^. The Oxygen Reduction Reaction (ORR) occurring at ODCs serves as a cornerstone for effective energy conversion and storage processes. Understanding the intricacies and nuances of ORR in ODCs is paramount for elevating device performance, longevity, and overall efficiency^5^. Moreover, ORR plays a pivotal role in oxygen-depolarized electrolyzers, where it drives the conversion of oxygen into hydroxide ions using electrical energy. Addressing the challenges associated with ORR in ODCs demands interdisciplinary collaboration, uniting expertise from fields such as materials science, electrochemistry, and device engineering^6,7^. Through the integration of recent advancements and the exploration of novel research avenues, the development of efficient, cost-effective, and sustainable ORR-based ODCs holds tremendous potential for propelling the realm of electrochemical energy conversion forward, thus contributing to a cleaner and more sustainable energy landscape^8–13^.

ORR has sluggish kinetics in both acidic and alkaline media, and the recent research focused on ORR electrocatalysts, including platinum group metal (PGM), non-PGM, carbon-based materials, and single-atom catalysts^14–19^. Alongside experimental works, the computational approaches were frequently applied to evaluate the ORR on platinum-based catalysts and other metals^20–25^. Nørskov et al*.*^26^ explained that Pt is the best metal catalyst among different transition and noble metals for ORR in acidic media by a volcano relationship between the rate of the cathode reaction and the intermediates binding energy (*O or *OH). Zhang et al*.*^27^ systematically studied the ORR at various Pt–Bi surfaces by DFT calculations. Their results showed that the introduction of Bi into Pt changes the potential-determining step (RDS) of ORR and PtBi (100) structure, with the lowest d-band center, gives the best ORR activity compared to the Pt (111). Gao^28^ explored the mechanism of the ORR on M–N_3_ (M = Mn, Co, Ni) co-doped defective graphene. At pH = 0, the overpotentials of ORR for Mn-N_3_-Gra, Co-N_3_-Gra, and Ni-N_3_-Gra are 0.56 V, 0.849 V, and 0.381 V, respectively. These results indicated that Ni-N_3_-Gra is comparable with Pt. It is also to be mentioned that most of the computational investigations of the ORR have concentrated on acid solutions and there are few theoretical studies have been conducted on alkaline conditions. As an example of studying in an alkaline environment, we can refer to Yu et al.^29^. They have studied two ORR mechanisms, i.e., dissociative and associative mechanisms, over different N-doped graphene on the cathode of fuel cells by DFT calculations. In this study, it can be seen that the ORR path through the associative mechanism is more favorable than the dissociative one by comparing the free energy curves. The results indicated the rate-determining step is the removal of *O from the surface of the N-doped configuration. Their results illustrated that this ORR catalyst is promising to replace Pt in the alkaline medium. Ignaczak et al*.*^30^ proposed a complete reaction sequence of ORR in alkaline solutions by comparing the adsorption of O_2_ on three metals such as Au(100), Ag(100), and Pt(100). According to the first step in ORR (*O*_*2*_ + *e*^*−*^* → *${O}_{2}^{-}$), they claimed that a small adsorbate cannot exist in two diverse charge states on a surface. Likewise, the reaction rate on a metal surface cannot be affected by the electrode potential. So at least one of the reactants must be in the outer sphere. Due to the strong electronegativity of oxygen, it is adsorbed in the form of the ${O}_{2}^{-}$ on Ag (100), and both the inner sphere and the outer sphere mechanism can occur in parallel. About Au (100), the first step of ORR takes place in the outer sphere mode, and on Pt (100), the adsorbed state is favorable. They have investigated that either reaction follows a 4e^−^ or 2e^−^ mechanism, depending on the adsorption energy of OH.

In order to properly interpret experimental results and predict the properties of new materials before synthesis, a detailed analysis by DFT calculations is needed. In our previous study, we conducted an in-depth analysis of the oxygen reduction reaction mechanism in alkaline media by DFT calculations, with a particular emphasis on single atoms of non-precious metals^31^. Through examination of Gibbs free energy profiles and the electrochemical-step symmetry index (ESSI), it was revealed that the SAC containing copper exhibited the most remarkable catalytic activity. This study is aimed to explore the ORR mechanism on single metal (Fe and Cu) and dual metal atom catalysts (Fe–Cu) supported on N-doped graphene considering solvent such as water (ε = 78.54) and the gas phase (ε = 1). This theoretical research in the gas and liquid phases can be a critical step towards understanding the complexity of cathodic reaction in Electrochemical devices and can be used as a guide for further studies.

## ORR mechanism in alkaline media

The focus of this study is on the associative mechanism of ORR under alkaline media where O_2_ proceeds to OH^−^ on the surface through four reaction steps as follows:

1 $$O_{2} + * + H_{2} O + e^{ - } \to {}^{*}OOH + OH^{ - } ,$$

2 $${}^{*}OOH + e^{ - } \to {}^{*}O + OH^{ - } ,$$

3 $${}^{*}O + H_{2} O + e^{ - } \to {}^{*}OH + OH^{ - } ,$$

4 $${}^{*}OH + e^{ - } \to OH^{ - } + *,$$

which involves the *O, *OH, and *OOH intermediates. We followed the mechanism from Liang et al.^32^ to compute Gibbs free energy change (Δ*G*_*i*_, *i* = 1–4) for each step and predict the theoretical overpotential for both gas and solvent phases. ΔGi values are calculated as follows:

5 $$\Delta G_{1} = \left[ {G\left( {*OOH} \right) + G\left( {OH^{ - } } \right)} \right]{-}\left[ {G\left( {O_{2} } \right) + G\left( * \right) + G\left( {H_{2} O} \right) + G\left( {e^{ - } } \right)} \right],$$

6 $$\Delta G_{2} = \left[ {G\left( {*O} \right) + G\left( {OH^{ - } } \right)} \right]{-}\left[ {G\left( {*OOH} \right) + G\left( {e^{ - } } \right)} \right],$$

7 $$\Delta G_{3} = \left[ {G\left( {*OH} \right) + G\left( {OH^{ - } } \right)} \right]{-}\left[ {G\left( {*O} \right) + G\left( {H_{2} O} \right) + G\left( {e^{ - } } \right)} \right],$$

8 $$\Delta G_{4} = \left[ {G\left( * \right) + G\left( {OH^{ - } } \right)} \right]{-}\left[ {G\left( {*OH} \right) + G\left( {e^{ - } } \right)} \right].$$

According to the literature^33,34^, G* is the total energy of the clean catalyst model, so G(*) = E*, whereas for the intermediates (X = *OOH, *O, *OH) on the surface, we have:

9 $$G\left( {\text{X}} \right) = E\left( {\text{X}} \right) + E_{{{\text{ZPE}}}} \left( {\text{X}} \right){-}TS\left( {\text{X}} \right),$$

where E(X) represents the total energy of the model with the adsorbed species X, EZPE(X) is the vibrational zero-point energy, and S(X) stands for the entropy of the adsorbed species. To calculate the only remaining terms *G(OH*^−^*) − G(e*^−^*)*, we follow Liang et al*.* method which used the equilibrium as *H*_*2*_*O(l) → H*^+^  + *OH*^−^, for which it holds that *G(OH*^−^*)* + *G(H*^+^*)* = *G(H*_*2*_*O(l))*, adding and subtracting *G(e*^−^*)* in the left-hand side one leads to^32^

10 $$G\left( {OH^{ - } } \right) - G\left( {e^{ - } } \right) = G\left( {H_{2} O} \right){-}\left\{ {G\left( {H^{ + } } \right) + G\left( {e^{ - } } \right)} \right\} = G\left( {H_{2} O} \right) - 1/2G\left( {H_{2} } \right).$$

Substituting Eq. (10) into Eqs. (5)–(8), one gets

11 $$\Delta G_{10} = G\left( {*OOH} \right){-}\left[ {G\left( {O_{2} } \right) + G\left( * \right) + G\left( {H_{2} O} \right)} \right] + G\left( {H_{2} O} \right) - 1/2G\left( {H_{2} } \right),$$

12 $$\Delta G_{11} = G\left( {*O} \right){-}G\left( {*OOH} \right) + G\left( {H_{2} O} \right) - 1/2G\left( {H_{2} } \right),$$

13 $$\Delta G_{12} = G\left( {*OH} \right){-}\left[ {G\left( {*O} \right) + G\left( {H_{2} O} \right)} \right] + G\left( {H_{2} O} \right) - 1/2G\left( {H_{2} } \right),$$

14 $$\Delta G_{13} = G\left( * \right){-}G\left( {*OH} \right) + G\left( {H_{2} O} \right) - 1/2G\left( {H_{2} } \right).$$

In addition, applying an electrochemical-thermodynamic approach^35^, we can introduce the potential relative to the SHE and the pH in Eqs. (11)–(14), then

15 $$\Delta G\left(pH,U\right)=\Delta G\left(\mathrm{0,0}\right)-v\left({H}^{+}\right){k}_{B}T({\text{ln}}10)pH--v\left({e}^{-}\right)eU,$$

where v(H^+^) and v(e^−^) are the numbers of transferred protons and electrons, k_B_ is the Boltzmann constant, e is the elementary charge of an electron, and U is the applied electrode potential with respect to the SHE^36^. It is also necessary that we consider thermodynamic limiting potential (U_L_) for a real catalyst. U_L_ is the highest potential at which all reaction steps are exergonic^37^ and is defined as

16 $$U_{{\text{L}}} = U_{0} - \frac{1}{e}[max(\Delta G_{i} )_{i \, = \, 10 - 13} ].$$

The theoretical overpotential (ƞ_th_) serves as an indicator of the effectiveness of a catalyst activity, which is defined as the difference between the equilibrium potential (U_0_) and the limiting potential (U_L_), this is

17 $$\eta_{th} = U_{0} - U_{L} .$$

U_0_ for ORR in alkaline media is equal to + 0.4 V vs. SHE. It is also important to note that for the ideal catalyst, all steps have the same ΔG_i_, and U_L_ = U_0_ leading to ƞ_th_ = 0.

## Computational details

### Methods

All our calculations, in DFT framework, were carried out in Dmol3 code embedded in the Material studio^38,39^. To describe the exchange–correlation function, the generalized gradient approximation (GGA) with Perdew-Burke-and-Ernzerhof (PBE) function was used^40^. DFT Semi-core Pseudopotentials (DSPP) were employed to achieve valid results, and double numerical plus polarization (DNP) basis sets were chosen^41^. For describing the van der Waals type long-range forces between absorbent species and host surface, the approach of Grimme was used^42^. For the unit cell of 5 × 5 × 1 with lattice parameters (a = b = 12.3 Å, c = 20 Å), a 4 × 4 × 1 Monkhorst–Pack k-point sampling was utilized. The convergence tolerances of energy and smearing in the geometric optimization process were 1 × 10^−5^ Ha and 0.005 Ha, respectively. The conductor-like screening model (COSMO) is used to simulate the aqueous environment, where the dielectric constant was set as 78.54 (H_2_O)^43^.

### Models

**Figure 1:**
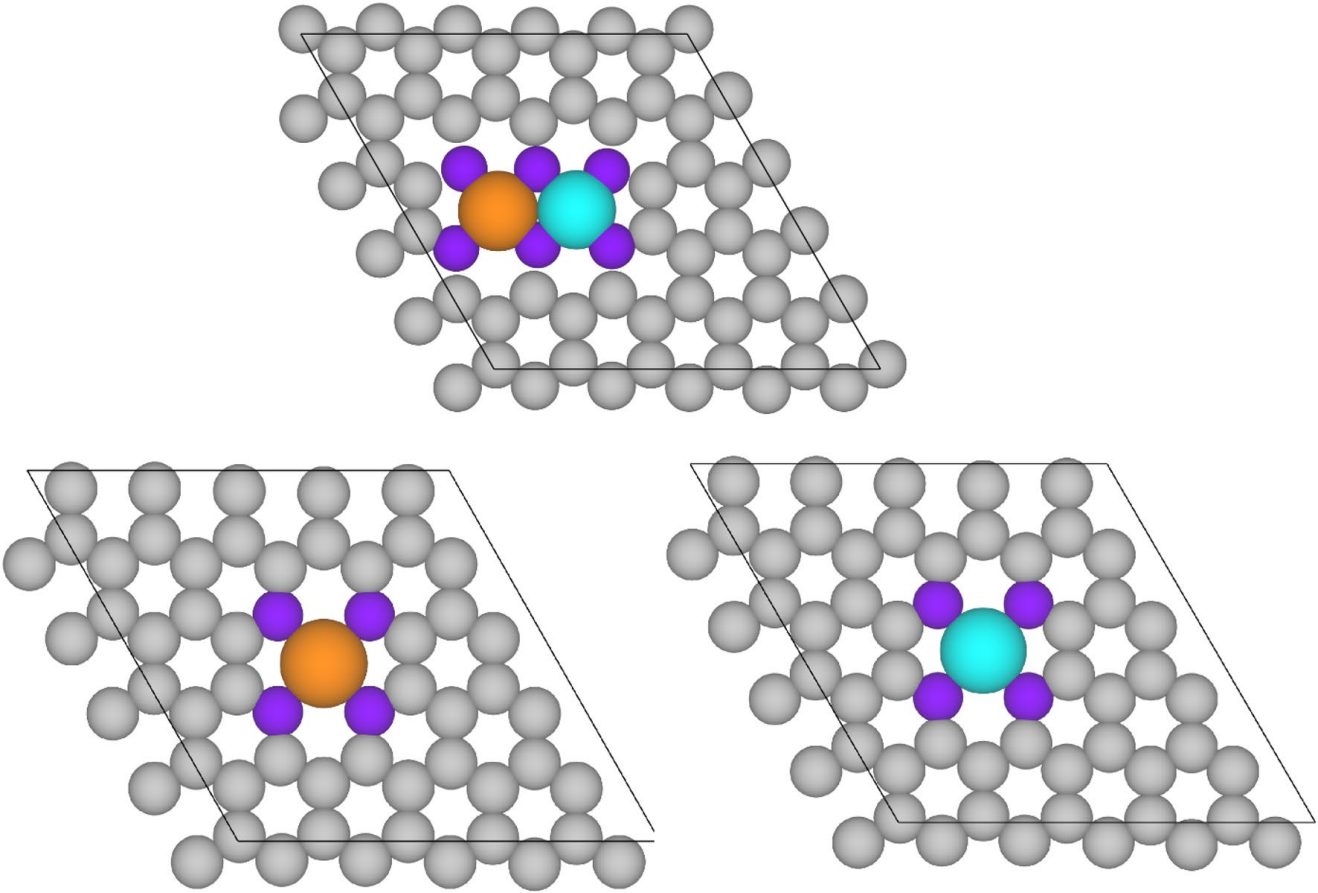
Schematic representation of the model of single and dual atom electrocatalysts in the present work. Grey, purple, orange, and light blue balls denote C, N, Cu, and Fe, respectively.

We consider a 5 × 5 × 1 supercell for graphene where two single metal atoms (Fe and Cu) are coordinated to six N atoms as indicated in Fig. 1. In this way, the metal atoms form two six- and one five-membered rings around them and one four-membered ring together. The final unit cell contains 40 C atoms, 6 N atoms, and 2 single metal atoms. To prevent interaction between periodic replicas, in periodic calculations, the 20 Å value is chosen as the third lattice parameter (c). In this study, the results are compared with the electrode model which includes one single atom (Cu or Fe) in the structure of a graphene supercell and four N atoms. The metal atom forms two five- and two six-membered rings (Fig. 1).

## Results and discussion

### Adsorption energy and electronic properties

Adsorption energy is a measure of how strongly an adsorbate binds to the surface of a catalyst. DFT calculations can provide valid information about the electronic and geometric properties of adsorbates (*O_2_, *OOH, *O, *OH) and substrates. First, we need to optimize the model and do the DFT calculations for three different cases: (1) the isolated surface of catalyst in the supercell, (2) the isolated adsorbate molecules, and (3) the combined surface + adsorbate system.

The DFT calculations will give us the total energies of each case, which we can use to calculate the adsorption energy using this formula:

18 $${\text{E}}_{{{\text{ads}}}} = {\text{E}}_{{{\text{System}}}} {-}\left[ {{\text{E}}_{{{\text{Surface}}}} + {\text{E}}_{{\text{X}}} } \right].$$

where E_system_ is the total energy of the optimized system, E_Surface_ is the total energy of the bare surface and E_X_ is the total energy of an isolated X adsorbate (X = *O_2_, *OOH, *O, *OH). A more negative the E_ads_ illustrates a better thermodynamically favorable adsorption process^44^. Table 1 summarizes the obtained values including the adsorption energy of different optimized structures.

**Table 1:** Adsorption energy (*E*_*ads*_ in eV) of *O_2_, *OOH, *O, and *OH on different catalysts.

Catalysts	*O_2_	*OOH	*O	*OH
	Without solvent correction			
Fe	−0.48	−1.38	−2.53	−2.38
Cu	−0.98	−1.84	−4.48	−2.92
Fe–Cu	−0.82	−1.56	−2.66	−2.53

	With solvent correction			
Fe	−0.61	−2.43	−2.76	−1.38
Cu	−1.29	−3.05	−3.32	−2.11
Fe–Cu	−1.11	−2.82	−3.11	−1.90

In the model of the single-atom catalyst, a single metal (Fe or Cu) is coordinated by four nitrogen atoms in the graphene plane. The average distances between the metal and nitrogen atoms are 1.89 Å and 1.92 Å for the Fe@NC and Cu@NC structures, respectively. Due to the presence of a vacuum defect, the bond lengths of all of M–N are longer than the C–C (1.42 Å) in pristine graphene, consistent with previously reported results^45–47^. Moreover, the average bond length of M–N in this study is smaller than the reported M–N bond length in the M-N_3_-doped graphene, with a range of 1.93–2.45 Å^28^. These results indicate that the covalent bonds between the single metal atom and the nitrogen atoms are stronger than the reported structure of Ref.^28^. For dual atoms catalyst (Fe-Cu@NC), the average bond length of Fe–N and Cu–N are 1.70 Å and 1.78 Å, respectively. Consequently, the bond strength in the structure of Fe-Cu@NC is stronger than Fe@NC and Cu@NC.

The electronic character is a critical parameter that directly affects the electrochemical performance of a catalyst. Therefore, we investigated this parameter by density of states (DOS) and projected density of states (PDOS) plots. The results illustrated the metallicity of our structures (Fig. S1). According to these results, there are overlapped electronic states (M3d-N2p: M = Fe and Cu) due to the strong covalent bonding between single metal atoms and four nitrogen atoms. The asymmetric DOS profiles at the Fermi level (zero) and around it, indicate a strong magnetic moments for our catalytic models. Moreover, the strong interaction between single atom-substrate can effectively tune the electronics of SAC. In addition, The d-band centers of Fe@NC, Cu@NC, and Fe-Cu@NC were −3.83, −4.19, and −2.39 eV, respectively (Fermi level = 0). Based on the PDOS analyses and d-band theory, an upshift of the d-band center for the electrocatalysts is attributed to the charge transfer between the metal sites. The d-band of Fe-Cu@NC shifts close to Fermi level, and enhances the capture of the O_2_ molecule that is the initial step of ORR^47^. The band gap refers to the energy difference between the highest occupied molecular orbital (HOMO) and the lowest unoccupied molecular orbital (LUMO) in a material. Therefore, a catalyst with a smaller band gap may be more effective for reactions that involve electron transfer steps. The band structure graphs in Fig. S2 show the band gaps of Fe@NC, Cu@NC, and Fe-Cu@NC are 0.074, 0.447, and 0.033 eV, respectively. However, the choice of catalyst should not be solely based on the band gap. Other factors such as the specific reaction mechanism, the nature of reactants and intermediates, surface properties, and catalyst stability should also be considered.

Electron transferring has a key role in the performance of ORR electrocatalysis and the adsorption of O_2_. In this study, the useful Bader's theory of atoms in molecules is used for analyzing the charge. The assumption of maximum charge density in atomic centers (or at pseudoatoms) is considered in Bader's analysis^48,49^. In the ORR, firstly oxygen molecules adsorbed to the catalyst and gain electrons. In contrast, single metal atoms are the electron donor in this process. The average charge transfer for Fe and Cu are 1.08 and 0.90, respectively. This confirms that single metal atoms are suitable active sites that lead to considerable differences in charge transfer between substrates and oxygen molecules. The average charge transfers for C and N in all catalytic models are 0.09 and − 1.18, respectively. This confirms that, in addition to the single metal atoms, nitrogen atoms have also played an effective role in charge transfer in the oxygen reduction process^31^. In the case of Fe-Cu@NC, the average charge transfer for Fe and Cu are 1.12 and 0.91, respectively. The findings indicate that the presence of Fe sites could promote the adsorption or activation of O_2_ molecules, thereby indirectly affecting the behavior of intermediates on Cu sites. In essence, taking into account interactions with both Fe and Cu sites offers a more comprehensive insight into the catalytic mechanism^50^.

### Free energy

**Figure 2:**
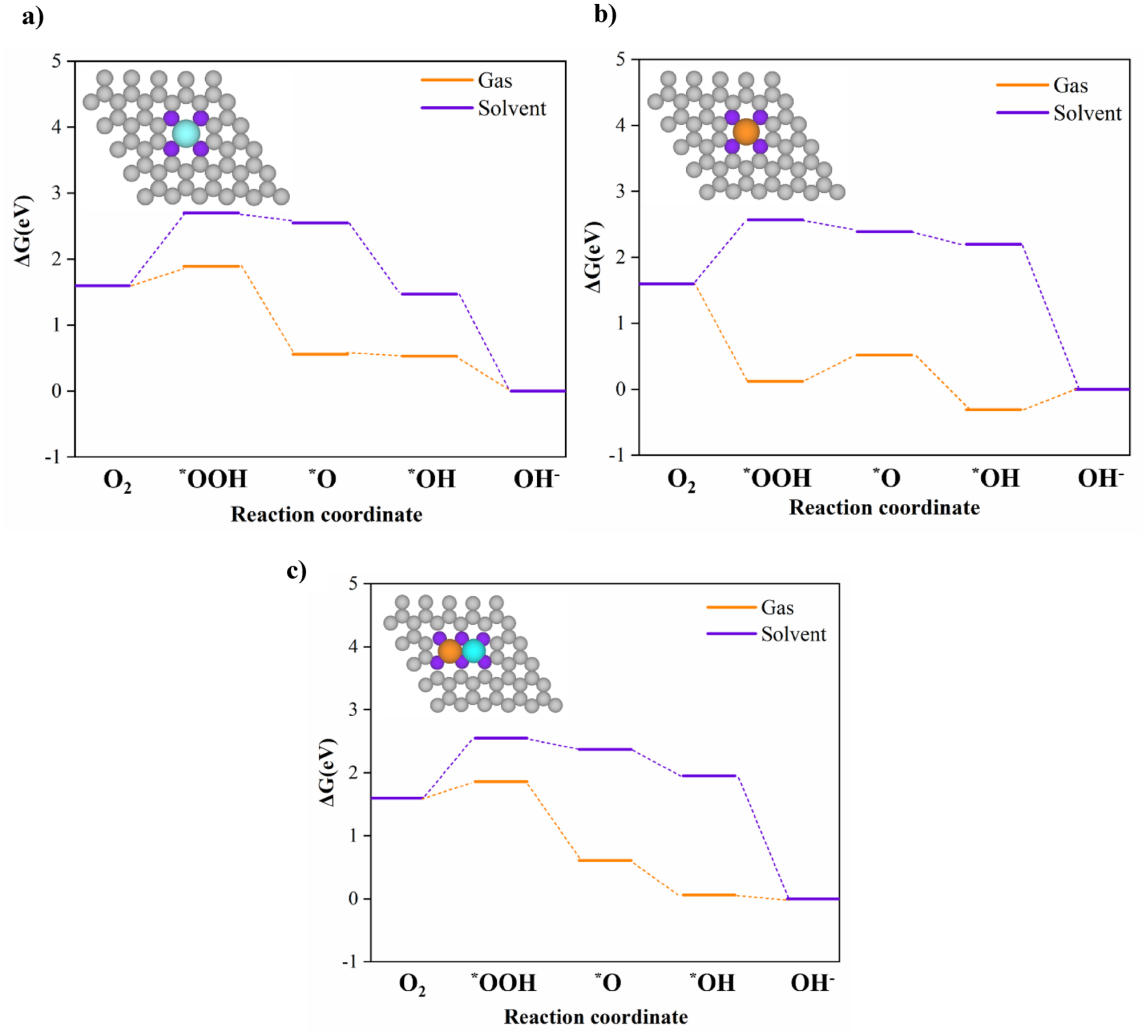
Gibbs free energy diagrams of ORR in gas and solvent phases for: (**a**) Fe@NC, (**b**) Cu@NC, (**c**) Fe-Cu@NC models considered at *U* = 0 V and *pH* = 14.

**Figure 3:**
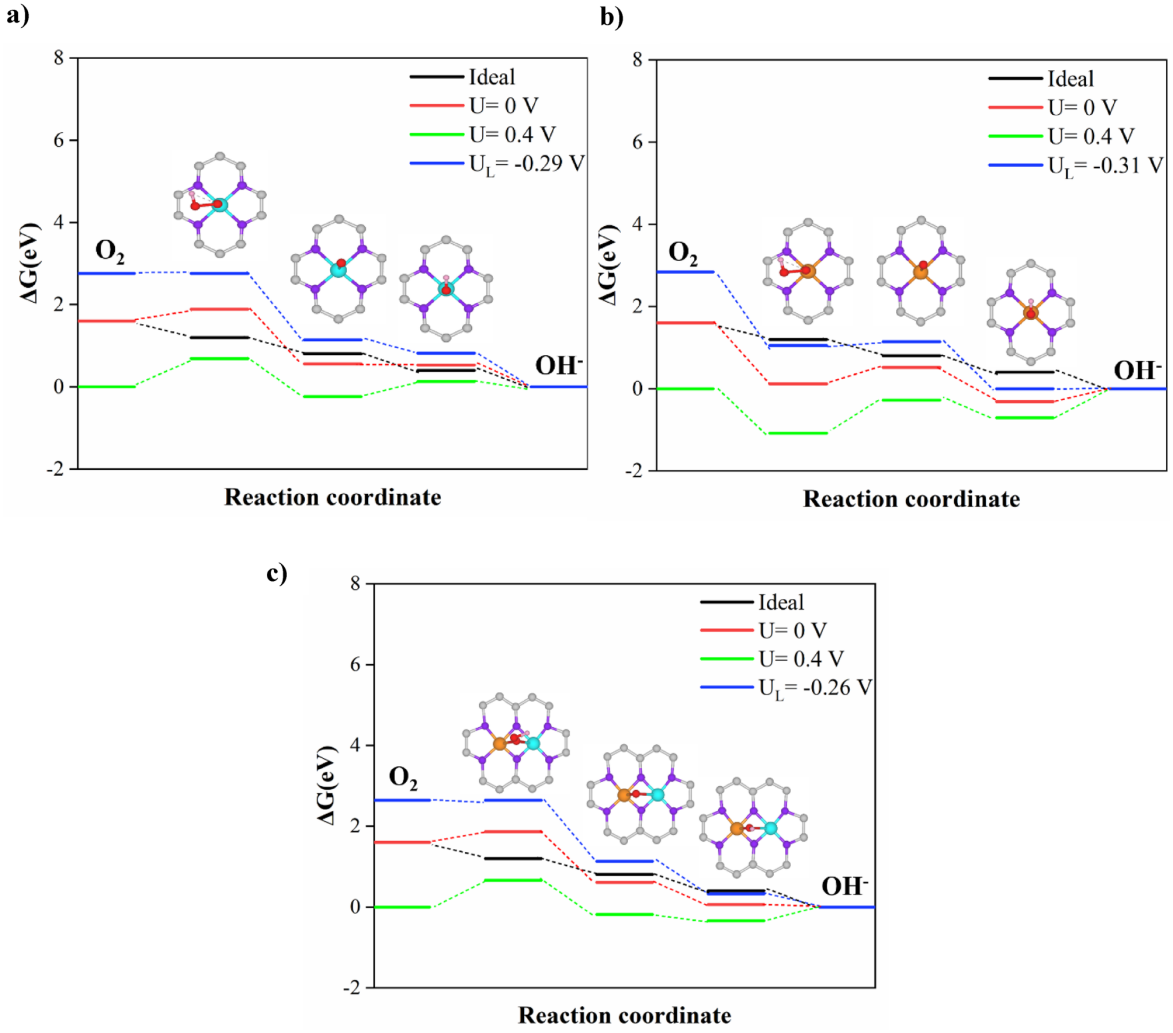
Gibbs free energy diagrams of ORR in the gas phase for: (**a**) Fe@NC, (**b**) Cu@NC, (**c**) Fe-Cu@NC models considered at different electrode potentials and *pH* = 14, at the equilibrium potential (U_0_ = 0.4 V) and at *U*_*L*_ (V *vs.* SHE), which is system dependent.

In this work, we considered the four-electron pathway for ORR in alkaline conditions to determine which catalyst has a lower overpotential (η_ORR_). First, we performed the calculations for two gas and solvent phases to evaluate the reaction path on different electrocatalysts. For the ideal catalyst at U = 0.4 V vs. SHE, the four steps have ΔG_i_ = 0 and η = 0. The rate determination step (RDS) with a positive ΔG value is thermodynamically difficult to occur and leads to a high overpotential^50,51^. According to Fig. 2 and Table 2, *OOH formation is the most difficult step to occur on Fe@NC and Fe-Cu@NC in two gas and solvent phase systems. In contrast, the formation of *O from *OOH and the formation of *OOH are the rate determination steps for Cu@NC in the gas and solvent phases, respectively. In addition, Fig. 3 shows the free energy change of all catalysts at different potentials and pH = 14, according to Eqs. (11)–(14) in the gas phase. According to Eq. (16), the calculated U_L_ values for Fe@NC, Cu@NC, and Fe-Cu@NC are −0.21, −0.31, and −0.26 V, vs. SHE, respectively. Also, the calculated overpotential values from Eq. (17) for Fe@NC, Cu@NC, and Fe-Cu@NC in the gas phase are 0.69 V, 0.71 V, and 0.66 V, respectively. Figure S3 shows the free energy change of all catalysts at different potentials and pH = 14, according to Eqs. (11)–(14) in the solvent phase. The calculated U_L_ values Fe@NC, Cu@NC, and Fe-Cu@NC are −1.35, −1.40, and −1.17 V, vs. SHE, respectively. The calculated overpotential values of Fe@NC, Cu@NC, and Fe-Cu@NC in the solvent phase are 1.75 V, 1.80 V, and 1.57 V, respectively. Therefore, the results in the solvent phase lead to a higher overpotential and thermodynamic limiting potential. It is also clear that the ORR kinetic offered by Fe-Cu@NC is suitable and has been identified as the best candidate among these electrocatalysts, which also showed the major impression resulting from the carbon substrate and N doping.

**Table 2:** Calculated Gibbs free energies (*ΔG*_*i*_, *i* = 1–4, in eV) in gas and solvent phases, the overpotentials (*η*), and the limiting potentials (*U*_*L*_) of different catalysts at *U* = 0 V, *pH* = 14.

Catalyst	*ΔG*_*1*_	*ΔG*_*2*_	*ΔG*_*3*_	*ΔG*_*4*_	*η*_*theo*_* (V)*	*U*_*L*_*(V)*
Ideal	− 0.40	− 0.40	− 0.40	− 0.40	0.00	0.00

	Without solvent correction					
Fe	0.29	−1.33	−0.03	−0.53	0.69	−0.29
Cu	−1.48	0.40	−0.83	0.31	0.71	−0.31
Fe–Cu	0.26	−1.25	−0.55	−0.06	0.66	−0.26

	With solvent correction					
Fe	1.10	−0.15	−1.08	−1.47	1.75	−1.35
Cu	0.97	−0.18	−0.19	−2.20	1.80	−1.40
Fe–Cu	0.95	−0.18	−0.42	−1.95	1.57	−1.17

## Conclusion

To increase the activity and performance of M–N/C catalysts, different strategies such as single-atom catalysts (SACs) and dual-atom catalysts can be used. Density functional theory calculations were employed to investigate the ORR activity of low-cost single Fe and Cu-doped and co-doped graphene-based structures in alkaline media as electrocatalysts of Oxygen-depolarized cathodes (ODCs) in advanced chlor-alkali electrolysis. In these catalytic models, the metal atoms are anchored at the hollow sites of N-doped graphene and each of them is coordinated to four nitrogen atoms. The four-electron ORR mechanism is investigated in the gas and solvent phases. The Gibbs free-energy profiles for different catalysts show that the results in the solvent phase lead to a higher overpotential and thermodynamic limiting potential. Among studied catalysts, Fe-Cu@NC is predicted to be the best candidate for cathode with the low thermodynamic limiting potential and theoretical overpotential of  −0.26 and  0.66 V vs. SHE, respectively, at pH = 14 in the gas phase. The results indicated that the bimetallic synergy between Fe and Cu in dual atom-doped graphene facilitated the ORR in alkaline media. In fact, DFT calculations in this work helped us to investigate the reaction mechanism for single-atom and dual-atom catalysts, estimate the adsorption energies of chemical species, determine the activation energy barriers of the reactions, and provide information about the electronic structure. This study can be a guide for the design and discovery of new catalysts for oxygen reduction reaction in alkaline media in the future.

## Supplementary Information

Supplementary Figures.

## Data Availability

Data is provided within the manuscript or supplementary information files.
