# Supplementary material for: Theoretical investigation of electrocatalytic activity of Pt-free dual atom-doped graphene for O2 reduction in an alkaline solution
Source: Sci Rep. 2024 Jun 20;14:14201. doi: 10.1038/s41598-024-61223-y (PMC11637109; doi:10.1038/s41598-024-61223-y)
Supplement: Supplementary file 1 — Supplementary Figures. [file 41598_2024_61223_MOESM1_ESM.docx]

Supporting File

**Theoretical investigation of electrocatalytic activity of Pt-free dual atom-doped graphene for O_2_ reduction in an alkaline solution**

**Tahereh Jangjooye Shaldehi, Soosan Rowshanzamir^*^**

*Hydrogen & Fuel Cell Research Laboratory, School of Chemical, Petroleum and Gas Engineering, Iran University of Science and Technology, Narmak, Tehran 16846-13114, Iran*

*^*^Corresponding author E-mail:* [*Rowshanzamir@iust.ac.ir*](mailto:Rowshanzamir@iust.ac.ir)

**Figure S1.** Total and PDOS diagrams of: a) Fe@NC, b) Cu@NC, c) Fe-Cu@NC models. Energy levels are referred to the Fermi energy.

**a)**


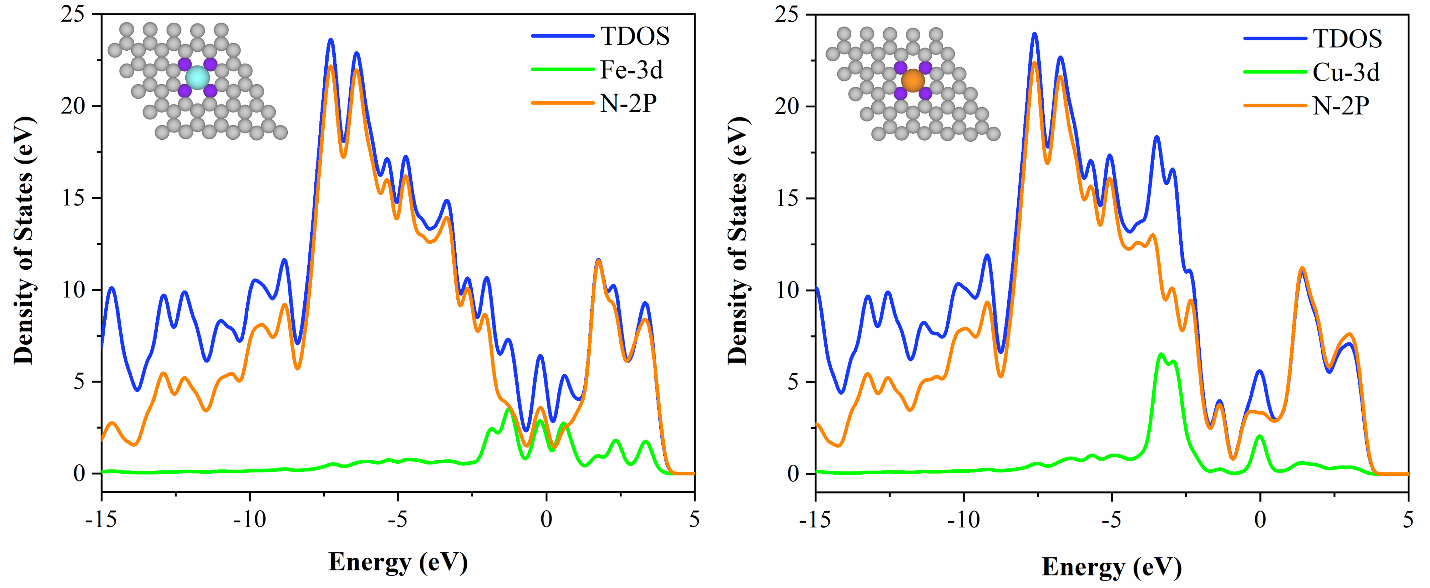


**b)**


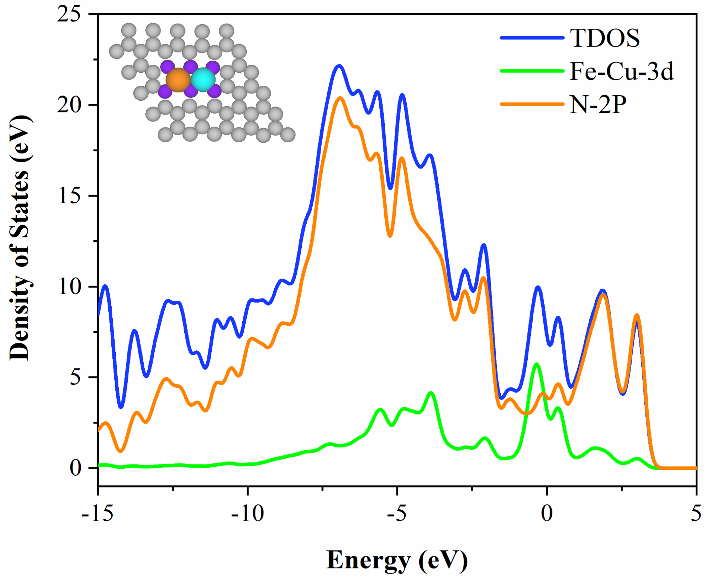


**c)**

**Figure S2.** Band structure diagrams of: a) Fe@NC, b) Cu@NC, c) Fe-Cu@NC models. Energy levels are referred to the Fermi energy.

**a)**

**b)**

**
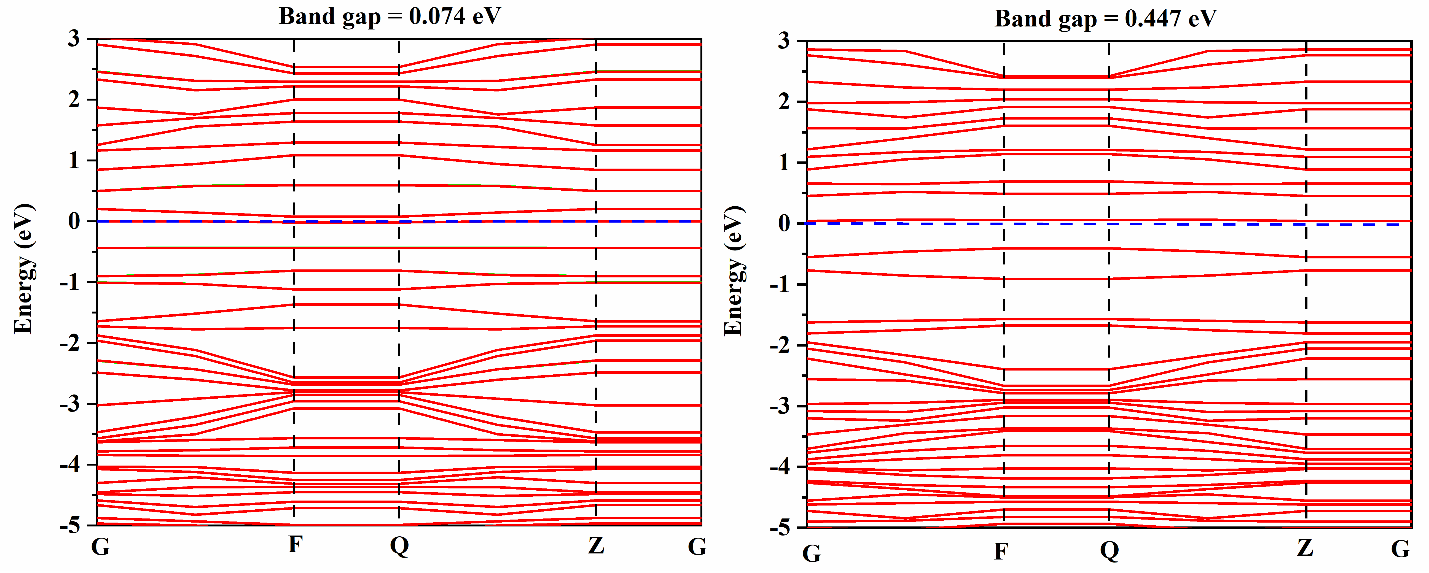
**

**c)**

**
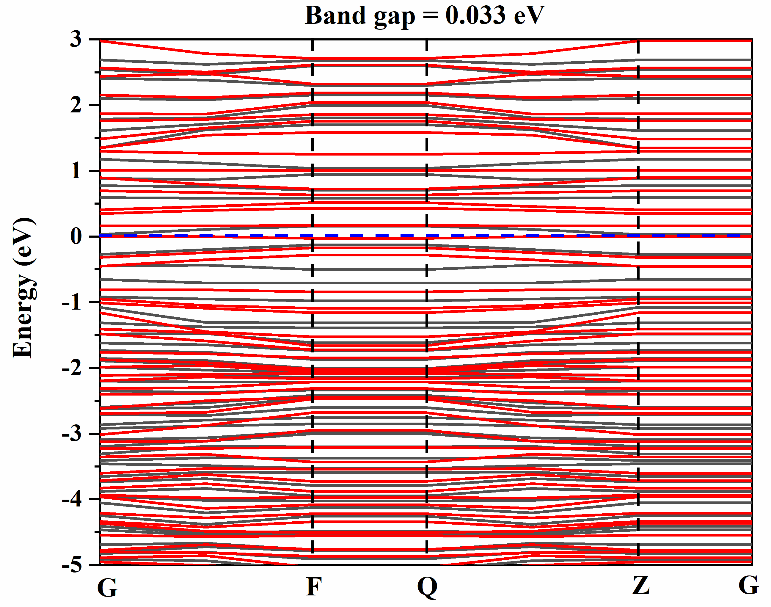
**

**Figure S3.** Gibbs free energy diagrams of ORR in solvent phase for: a) Fe@NC, b) Cu@NC, c) Fe-Cu@NC models considered at different electrode potentials and *pH* = 14.

**a)**

**b)**

**
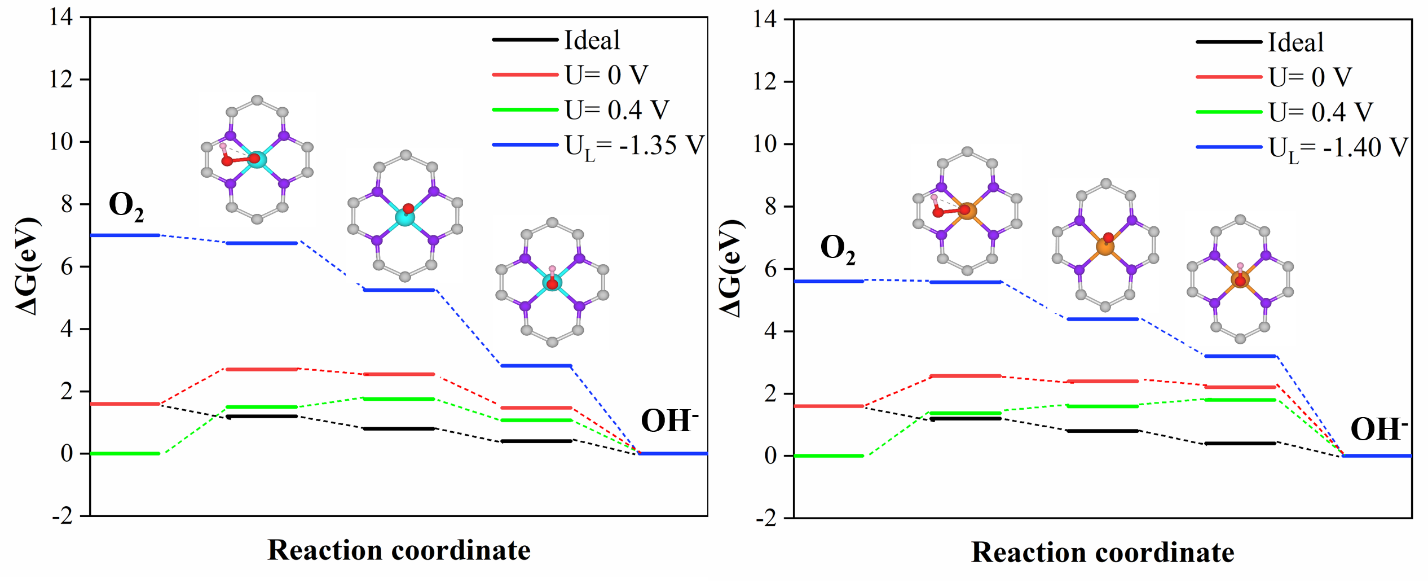
**

**
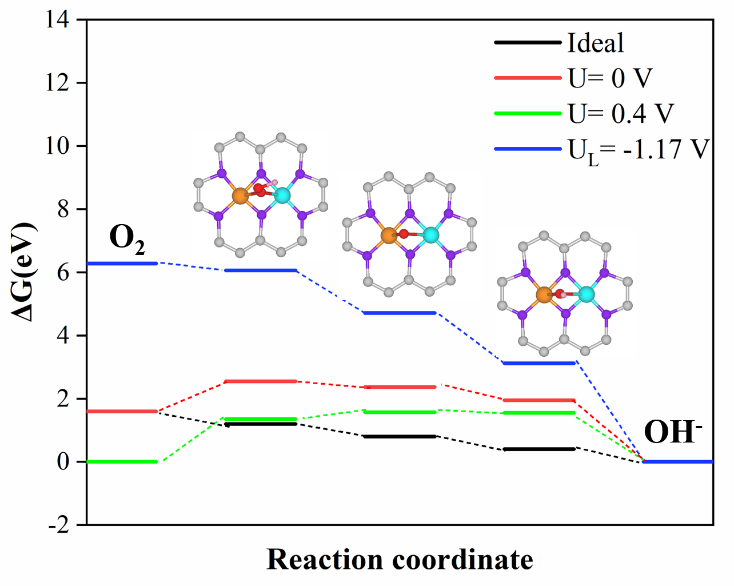
**

**c)**
